# Validation of a maternal questionnaire on correlates of physical activity in preschool children

**DOI:** 10.1186/1479-5868-6-81

**Published:** 2009-12-02

**Authors:** Alison M McMinn, Esther MF van Sluijs, Nicholas C Harvey, Cyrus Cooper, Hazel M Inskip, Keith M Godfrey, Simon J Griffin

**Affiliations:** 1 MRC Epidemiology Unit, Institute of Metabolic Science, Addenbrooke's Hospital, Hills Road, Cambridge, CB2 0QQ, UK; 2 MRC Epidemiology Resource Centre, Southampton General Hospital, Southampton, SO16 6YD, UK

## Abstract

**Background:**

Valid measures of physical activity correlates in preschool children are lacking. This study aimed to assess the validity, factor structure and internal consistency of a maternal questionnaire on potential correlates of four-year-old children's physical activity.

**Methods:**

The questionnaire was designed to measure the following constructs: child personal factors; parental support and self-efficacy for providing support; parental rules and restrictions; maternal attitudes and perceptions; maternal behaviour; barriers to physical activity; and the home and local environments. Two separate studies were conducted. Study I included 24 mothers of four-year-old children who completed the questionnaire then participated in a telephone interview covering similar items to the questionnaire. To assess validity, the agreement between interview and questionnaire responses was assessed using Cohen's kappa and percentage agreement. Study II involved 398 mothers of four-year-old children participating in the Southampton Women's Survey. In this study, principal components analysis was used to explore the factor structure of the questionnaire to aid future analyses with these data. The internal consistency of the factors identified was assessed using Cronbach's alpha.

**Results:**

Kappa scores showed 30% of items to have moderate agreement or above, 23% to have fair agreement and 47% to have slight or poor agreement. However, 89% of items had fair agreement as assessed by percentage agreement (≥ 66%). Limited variation in responses to variables is likely to have contributed to some of the low kappa values. Six questions had a low kappa and low percentage agreement (defined as poor validity); these included questions from the child personal factors, maternal self-efficacy, rules and restrictions, and local environment domains. The principal components analysis identified eleven factors and found several variables to stand alone. Eight of the composite factors identified had acceptable internal consistency (α ≥ 0.60) and three fell just short of achieving this (0.60 > α > 0.50).

**Conclusion:**

Overall, this maternal questionnaire had reasonable validity and internal consistency for assessing potential correlates of physical activity in young children. With minor revision, this could be a useful tool for future research in this area. This, in turn, will aid the development of interventions to promote physical activity in this age group.

## Background

The promotion of physical activity in children is a key public health issue due to its potential contribution to the prevention of obesity [1], osteoporosis [2], the metabolic syndrome [3] and cardiovascular disease[4] and its association with positive effects on mental health [2]. Recent evidence suggests that children and adolescents are not undertaking recommended amounts of physical activity [5,6], emphasising the need to develop ways of encouraging children to be more active. Evidence that physical activity tracks from early childhood [7,8] suggests that health behaviours (including activity) may develop in early life, making an argument for focusing promotion efforts on preschool children. However, the few intervention studies that have targeted this age group have not reported significant effects [9]. To develop effective interventions to promote physical activity in this age group, an understanding of the determinants or correlates of this behaviour is required but this is currently limited. Research into physical activity correlates in young children has mostly focused on demographic and biological factors [10] warranting further research across a range of domains.

An important aspect of research into the correlates of physical activity in children is the use of valid and reliable measures, both of physical activity and of the correlates being investigated. Using measures with acceptable levels of validity and reliability reduces measurement error and strengthens the conclusions that can be drawn. Studies have investigated the psychometric properties of measures of correlates in adolescents [11-17] and older children (aged 8-12 years) [18-22] but not preschool children. We therefore aimed to assess the psychometric properties of a maternal questionnaire designed to measure a range of factors that might be associated with preschool children's physical activity. The questionnaire was based on a literature search on the correlates of young (< 8 years) children's physical activity and guided by Social Cognitive Theory which identifies personal, behavioural and environmental (social and physical) influences on children's health behaviour [23]. Drawing on these, the questionnaire was designed to measure the following domains: child personal factors; parental support and self-efficacy for providing support; parental rules and restrictions; maternal attitudes and perceptions; maternal behaviour; barriers to physical activity; the home environment; and the local environment. Additionally, questions on whether or not the child attended preschool/nursery and what mode of transport mothers chose when making short trips were included to give some indication of the child's lifestyle.

The validation of measures of physical activity correlates in children is problematic, largely due to there being no established 'gold standard' with which to compare a new measure. Some validation studies of child-report questionnaires have used alternative sources of information, such as parents [11] or teachers [21], as the criterion measure for comparison. However, the accuracy of these criterion measures is unclear. Other validation studies have investigated different aspects of validity such as factorial validity [15,16,24], which is used to assess which questions contribute the greatest variation in responses and to detect the underlying structure of a set of variables. This can be used either to reduce a set of questions on a particular construct or to guide the grouping of individual variables into constructs for analyses. While this can provide useful information, on its own, it does not verify whether the new questionnaire is measuring what it is supposed to. Some studies have also looked at the relationship between measures of correlates and levels of children's physical activity to further validate questionnaires [17,19,20]. However, this seems counter-intuitive as it is investigating whether the variables in a questionnaire are associated with physical activity before it has been established whether the questionnaire is accurately measuring these variables. As there does not appear to be one ideal way to validate questionnaires on the correlates of physical activity in children, using a combination of methods is likely to give the best estimates of validity.

The aims of the studies presented here were to assess the validity of a maternal questionnaire on the potential correlates of physical activity among preschool children and to explore the factor structure of the questionnaire data, and the internal consistency of the factors identified, to guide future analyses with these data. To our knowledge, this is the first study to investigate the validity and reliability of a questionnaire on the potential correlates of physical activity in preschool children.

## Methods

### Questionnaire Development

The questionnaire was developed for use in the Southampton Women's Survey (SWS), a cohort study designed to investigate how women's anthropometry, lifestyle, and nutrition, before and during pregnancy, affect the development of their offspring [25]. Participants are monitored during their pregnancy then followed up after the birth of their child. When the children are four years old, a sub-sample is seen for a dual energy x-ray absorptiometry (DXA) scan [25], forming the source of participants for a secondary study investigating the association between bone health and obesity. This part of the SWS also aims to investigate the association between objectively measured physical activity and obesity and the correlates of physical activity. The questionnaire was therefore developed for this part of the study to collect data on the potential correlates of physical activity.

The questionnaire included both newly developed questions and previously used questions that have been associated with children's physical activity. The newly developed questions (which included new questions for previously investigated constructs) covered the following potential correlates: maternal self-efficacy for providing social support; method of transport used when making short trips; mother's perception of child's physical activity compared with siblings and peers; child's personality; and parental importance of child's physical activity. Questions that were derived from previous studies covered: maternal perception of the local environment [26,27], maternal physical activity and sedentary pursuits [28], parental support [29,30], physical activity rules [26], physical activity restrictions [26], parental concern about child's television watching [31], child enjoyment of physical activity [32], child's preference for physical activity [30], and barriers to child's physical activity [26]. As data on biological factors were already being collected in the SWS, questions on these factors were not included in the questionnaire. The questionnaire was initially reviewed by a group of ten researchers who had experience of working on research involving children, to gauge the appropriateness of the questions and identify any further questions that should be added. The questionnaire was then pilot tested with five mothers of preschool children. A few amendments to the questions were made following feedback from these stages. The final version of the questionnaire contained 83 individual items, grouped into 31 questions, and took around 15-20 minutes to complete. The analyses presented in this paper relate to 20 questions (consisting of 63 individual items). A description of the variables/constructs covered in these questions is given in Table 1. Questions not included in the analyses were those relating to demographics (e.g. ethnicity, parent's age), mothers' weight status, and mothers' sedentary behaviour and physical activity which had previously been validated [28,33].

**Table 1:** Description of questionnaire items/constructs

Name of variable/group of variables^1^	No. of items	Description/abbreviated questions^1^
**Child personal factors**		
Personality	4	Would you describe your child as: physically active; restless; well-behaved; inquisitive/outgoing (strongly disagree to strongly agree).
Enjoyment of physical activity	1	Agreement with: my child enjoys being physically active (strongly disagree to strongly agree).
Preference for physical activity	3	Does your child prefer to: play indoors or play outdoors; play with toys or watch TV; watch TV or play a running a game with siblings or friends.

**Parental support**		
Parental support	5	How often (never to very often) do you or your partner: encourage child to do PA; do PA with child; provide transport so child can go somewhere to do PA; watch child participate in PA; tell child PA is good for health?
Maternal self-efficacy for social support	7	Agreement (strongly disagree to strongly agree) with: I think it is difficult to: encourage child to go outside and play; encourage child to play active game instead of watching TV; play active game with child on a busy day; take child out to play when it's cold and wet; take child out to play when it's hot; play active game with child at the weekend; play active game with child when tired.

**Parental rules & restrictions**		
Parental rules	7	How often (never to very often) do you or your partner apply the following rules: not allowed to watch TV at meal times; go to bed at a set time; not allowed to play ball games in house; not allowed to eat snacks while watching TV; not allowed to play in playground without adult supervision; not allowed to run or cycle in house; not allowed to play in garden without adult supervision.
Parental restrictions	4	How often (never to very often) do you or your partner restrict the time child spends: watching TV/video; playing computer games; playing outside; using computer.

**Maternal attitudes & perceptions**		
Importance of child PA	1	Agreement with: I think it is important that my child participates in PA (strongly disagree to strongly agree).
Concern about TV watching	1	Agreement with: I am concerned about the amount of TV my child watches (strongly disagree to strongly agree).
PA compared to siblings	1	Compared to your other child(ren), would you say that your 4-year-old is: as active, more active, less active, not applicable (no other children).
PA compared to peers	1	Compared with children the same age and sex, would you say that your child is: generally less active; similarly active; generally more active.

**Barriers to physical activity**		
Perceived barriers	10	How often (never to very often) is child's PA limited due to: fees for clubs/swimming pools being too high; difficult to get to PA places; child lacks skills to do PA; child not interested in doing PA; weather is too bad; too busy; scared child will get hurt; no playgrounds near home; no other children to play with; no adult to supervise child whilst playing.

**Home environment**		
Household composition	4	How many children younger and older than 4-year-old live at home; do you live with the father of your 4-year-old; are there any other adults living in your home.
TV/PC in bedroom	3	Does child have: TV; video/DVD player; computer in bedroom (yes/no).
Car ownership	1	Does household have any cars/vans available for use (yes/no).

**Local environment**		
Perceived local environment	8	Agreement (strongly disagree to agree) with: heavy traffic on local streets; concerned about strangers; somewhere at home where child can go out and play; concerned about road safety; public transport is limited; playgrounds close to home where child can play; playgrounds at child's nursery; other children near home who child can play with.

**Lifestyle**		
Day care	1	Does child attend day care, nursery or preschool (yes, full time; yes, part time; no, he/she is at home with me; no, he/she is at home with other adult; other).
Transport for short trips	2	When making short trips (less than 1/2 mile) alone/with child, what form of transport do you usually use (public transport; car; walking; bicycling; other).

1. PA = physical activity; TV = television; PC = personal computer.

2. The questionnaire also included questions on demographic variables, parental weight status, and parental physical activity and sedentary behaviour but these were not included in the analyses for this paper.

### Study Design

Two separate studies were undertaken: Study I assessed the validity of the questionnaire and Study II explored the factor structure of the questionnaire to aid future analyses of the questionnaire variables. Study I was conducted on a small convenience sample (n = 24) during the first few months that the questionnaire was distributed to the SWS participants. To assess the validity of each question, we interviewed participants who had completed the questionnaire and used the interview data as a criterion measure with which to compare the questionnaire data. Conducting interviews with participants allowed them to clarify the meaning of the questions and expand on their answers, giving a richer response to the questions with which to compare their original questionnaire responses. Following initial feedback from Study I, some amendments were made to the questionnaire. The amended version of the questionnaire was then distributed in the SWS and it was this revised version that was used for Study II (a copy of this can be found in additional file 1). Study II (n = 389) was an exploratory factor analysis using principal components analysis (PCA). This analysis focused on the groups of questions in the questionnaire that were thought to reflect pre-determined constructs. Detailed information on the participants, data collection and statistical analyses for the two studies are described below.

### Study I: Validity Analysis

#### Participants and Data Collection

Participants for this study were primarily recruited from the SWS sub-sample attending the four-year visit (n = 20) with additional recruitment of mothers of four-year-old children (n = 4) from another study being conducted around the same time in Cambridgeshire, UK [34]. The inclusion of participants from the latter study was to help recruit a broader mix of people. Ethical approval for data collection in the SWS was obtained from Southampton and South West Hampshire Local Research Ethics Committee and in the Cambridgeshire physical activity study from the Cambridge Research Ethics Committee.

The 20 participants recruited from the SWS were given the questionnaire when they attended a study visit (between September 2005 and February 2006) and asked for their consent to be contacted regarding the validation of the questionnaire. The additional four participants from the Cambridgeshire study were recruited between June and September 2006. Participants received the questionnaire by post and were asked for their consent to be contacted about the validation study on collection of the completed questionnaires. Participants from both studies who consented to being contacted were telephoned by a researcher, within around six weeks (median (inter-quartile range): 6.1 (4.0 to 9.4) weeks), to further inform them about the study and invite them to participate in a telephone interview. The interviews were conducted using a semi-structured schedule designed to cover the same questions as the questionnaire, asked in a similar way. Participants were encouraged to elaborate on their answers and asked for feedback on the layout and phrasing of the questionnaire. Two pilot interviews were conducted before the main data collection and minor revisions were made to the interview schedule following this. Data from these pilot interviews were not included in the present analysis. After obtaining verbal informed consent (on tape), interviews were tape-recorded and fully transcribed. Participants' responses to each question were coded, blind to participants' questionnaire responses, by two researchers independently. For most questions, responses were coded as positive, neutral or negative, although other coding schemes were used for some questions where appropriate (e.g. yes/no). Consensus was reached by discussion in the case of disagreement.

Data on descriptive characteristics of participants' four-year-old children were recorded as part of the original studies they were participating in. In the SWS, height and weight were measured using Leicester height measures and Seca digital scales, respectively. In the Cambridgeshire study, height and weight were measured using a calibrated stadiometer and Tanita TBF-531 scales, respectively. Other descriptive data were gathered with the questionnaire.

#### Statistical Analyses

Analyses were conducted using Stata version 8.0 (StataCorp LP, College Station, Texas). Descriptive statistics for the sample were calculated for both child and parent characteristics using standard summary statistics as appropriate. The inter-rater agreement for the interview codings and the agreement between the interview codings (agreed on by both authors) and the questionnaire responses for each item were calculated using Cohen's kappa (for nominal categorical variables) or weighted kappa (for ordinal categorical variables). The level of agreement was categorised according to the cut-offs proposed by Landis & Koch [35] as poor (< 0.00), slight (0.00-0.20), fair (0.21-0.40), moderate (0.41-0.60), substantial (0.61-0.80), or almost perfect (0.81-1.00). As a limitation of Cohen's kappa is that it is dependent on the frequency of different responses [36], the percentage agreement between the interview and questionnaire responses was also calculated for each item. A cut-off of 66% was used to indicate fair agreement [18,37].

### Study II: Principal components analysis and internal consistency

#### Participants and Data Collection

Participants for this part of the analyses were those in the SWS sub-sample who attended a study visit between March 2006 and February 2008 and completed the amended version of the questionnaire (n = 398).

#### Statistical Analyses

Using Stata version 10 (StataCorp LP, College Station, Texas), a PCA was conducted on each group of variables in the questionnaire designed to reflect a particular construct. These included: children's personality and enjoyment of physical activity; child's physical activity preferences; parental support; maternal self-efficacy for social support; parental rules; parental restrictions; barriers to children's physical activity; and the local environment. For each analysis, factors with an eigenvalue less than 1.00 were dropped. Also, any individual variables that had a high percentage of variance (≥ 60%) unexplained by the factors that emerged were removed from the analysis as this suggested that the variable was not well explained by the common factors identified [38]. Variables that were found to have low validity in Study I and had not been revised for the amended version of the questionnaire were also excluded from the PCA.

Factor loadings for variables that remained in the analysis were rotated using varimax (orthogonal) rotation. Variables had to have a loading of 0.30 or greater to be assigned to a factor. For each factor that was identified, a Cronbach's alpha was calculated to assess the internal consistency of the factor. A Cronbach's alpha of ≥ 0.60 was used to indicate acceptable internal consistency [18,39].

## Results

### Study I Results

A total of 26 mothers consented to being contacted about the validation study, of which 24 were interviewed. Characteristics of the 24 participants are given in Table 2. The ethnic mix is similar to that of Southampton as a whole [40].

**Table 2:** Participant characteristics for Studies I and II

Variable^1^	Study I (n = 24)	Study II (n = 398)
Mother's age in years (Mean ± SD)	34.6 ± 4.0	35.0 ± 3.6
Mother's BMI (Median & IQR)	25.1 (22.3 to 29.1)	25.3 (22.5 to 29.4)
Child's BMI (Mean ± SD)	16.0 ± 1.1	16.2 ± 1.8
Gender of 4-year-old (% boys)	70.8	49.8
House-ownership (% own/buying house)	95.8	88.6
Age mother finished full-time education (Median & IQR)	19 (16 to 22)	18 (16 to 21)
Father lives in household (% yes)	100.0	90.7
Ethnicity (% white)	95.8	96.5

1. SD = standard deviation; BMI = body mass index; IQR = inter-quartile range.

The inter-rater agreement for the interview codings was substantial (kappa 0.75, 95% CI 0.72, 0.78). Table 3 shows the agreement between the questionnaire and interview responses. Three of the 63 questionnaire items assessed could not be given a kappa value due to the fact that all interview participants answered 'yes' to these questions, although all three had an agreement of 100%. Of the remaining 60 items, 18 (30%) had moderate agreement or above, 14 (23%) had fair agreement, and 28 (47%) had only slight or poor agreement according to kappa. However, 22 of the latter items had a percentage agreement of greater than 66% indicating that some of the low kappa scores were due to the lack of variation in responses rather than poor agreement. In total, six items (9.5%) had both a low kappa score (slight or poor) and low (< 66%) percentage agreement. These items related to: concern about stranger danger, rules on running and cycling in the house, restricting children's use of the computer, self-efficacy for taking children out to play when it is cold and wet and on a busy day, and children's preference for playing with toys or watching television. Two of these items - restricting computer use and rules on running and cycling in the house - were revised in time for Study II based on initial feedback from participants. It is believed that these revisions will have improved the validity of these questions. However, the four remaining variables with low validity were not revised and were therefore excluded from the PCA in Study II.

**Table 3:** Agreement between questionnaire and interview responses (criterion validity)

Name of factor^1^	Kappa	Percentage agreement	Items with low kappa^2 ^& low % agreement
**Child personal factors**			
Personality	-0.10-0.41 (poor-moderate)	65.2-83.3	None
Enjoyment of PA	-0.06 (poor)	87.0	None
Preference for PA	0.00-0.82 (slight-almost perfect)	56.5-94.4	Does child prefer to play with toys or watch TV

**Parental support**			
Parental support	0.04-0.28 (slight-fair)	75.0-80.4	None
Maternal self-efficacy for social support	-0.07-0.56 (poor-moderate)	50.0-87.0	Difficult to: take my child out to play when it's cold and wet; play an active game with my child on a busy day

**Parental rules & restrictions**			
Parental rules	0.00-0.47 (slight-moderate)	52.2-91.3	Not allowed to run or cycle in the house
Parental restrictions	0.05-0.59 (slight-moderate)	56.8-81.3	Restrictions on playing computer games/using the computer^3^

**Maternal attitudes & perceptions**			
Importance of child PA	-0.08 (poor)	88.6	None
Concern about TV watching	0.24 (fair)	78.3	None
PA compared to siblings	0.56 (moderate)	78.3	None
PA compared to peers	0.48 (moderate)	85.4	None

**Barriers to physical activity**			
Perceived barriers	-0.04-0.29 (poor-fair)	67.4-90.9	None

**Home environment**			
Household composition	All 1.00 except 1* (perfect)	All 100	None
TV/PC in bedroom	0.78-1.00 (substantial-perfect)	95.8-100	None
Car ownership	*	100	None

**Local environment**			
Perceived local environment	0.00-0.54 (slight-moderate)	62.5-93.8	I am concerned about 'stranger danger'.

**Lifestyle**			
Day care	*	100	None
Transport for short trips	0.29-0.52 (fair-moderate)	68.2-79.2	None

1. PA = Physical activity; TV = television

2. Low kappa defined as poor or slight agreement according to cut-offs by Landis & Koch [35].

3. For the criterion validity study, questions on whether parents restricted their child's computer use or computer game use were combined.

*Kappa could not be calculated due to the limited variation in response.

### Study II Results

399 participants received the new version of the questionnaire between March 2006 and February 2008. One asked to withdraw leaving 398 (99.7%) who returned a completed questionnaire. Characteristics for this sample are given in Table 2. The sample was mostly comparable to the interview sample but a lower proportion of mothers owned their home, lived with the father of their four-year-old and a lower proportion of the four-year-old children were boys. Comparisons of participant characteristics between those included in the analysis (n = 398) and those in the four-year sub sample who attended the visit prior to March 2006 (total n = 419) showed no significant differences in child's gender, child's age or mother's ethnicity.

Table 4 shows the results of the PCA with varimax rotation and the Cronbach's alpha for each factor identified. The questions regarding children's personality and enjoyment of physical activity were found to load onto two factors, with the exception of the question about whether mothers would describe their child as outgoing. This variable had a high unexplained variance by the two factors. No PCA was conducted on the child preferences construct as the removal of the question regarding children's preference for watching television or playing with toys due to its poor validity in Study I left only two variables within this construct. Together, these variables had low internal consistency (α = 0.27) suggesting that they do not represent the same construct. When these variables were added to a PCA with the personality and enjoyment variables, both were found to have a high unexplained variance, supporting the decision to treat these variables separately.

**Table 4:** Factor structure of pre-determined constructs in the questionnaire

Factor structure	**Variables**^1^	Eigenvalue	% variance explained	Cronbach's alpha
**Child personality/PA enjoyment**				
Factor 1	Describe child as physically active, child enjoys physical activity	1.62	40.5	0.76
Factor 2	Describe child as restless and as well-behaved (reversed)	1.37	34.3	0.51
*Variables removed*^2^*: describe child as outgoing*				
**Parental support**				
Factor 1	Encourage child to do PA; do PA with child; provide transport to places where child can do PA; watch child participate in PA	2.42	60.6	0.77
*Variables removed*^2^*: tell child PA is good for health*				
**Maternal self-efficacy for social support (Difficult to...)**				
Factor 1	Encourage child to go outside and play; encourage child to play active game instead of watch TV; play active game with child at weekend	1.86	61.9	0.68
*Variables removed*^2^*: take child outside to play when it is hot; play active game with child when feeling tired*				
**Parental rules (Child can...)**				
Factor 1	Watch TV at meal times; go to bed when they want to; eat snacks while watching TV	1.80	26.8	0.56
Factor 2	Play ball games in the house; run or ride a tricycle/scooter in the house	1.26	23.8	0.60
Factor 3	Play in the park/playground with older children	1.01	17.6	NA
*Variables removed: play in the garden without adult supervision*^3^				
**Parental restrictions**				
Factor 1	Restrict child's computer use; restrict child playing computer games	NA	NA	0.63
*Variables removed*^2^*: restrict child playing outside; restrict child's TV viewing*				
**Barriers to PA**				
Factor 1	Child is not interested in the activity; weather is too bad; I am too busy; scared child will get hurt	2.93	27.3	0.69
Factor 2	Fees for clubs/swimming pools are too high; difficult to get to PA places; no play areas/parks near home; no other children for child to play with	1.23	24.8	0.66
*Variables removed*^2^*: child lacks skills to do PA; no adult available to supervise child*				
**Local environment**				
Factor 1	Other children near home who child can play with; play areas, parks or gyms close to home where child can play	1.51	25.2	0.52
Factor 2	Heavy traffic on local streets; concerned about road safety in area	1.43	23.9	0.62
*Variables removed*^2^*: somewhere at home where child can play outside*^3^*; public transport is limited in our area*.				

1. PA: physical activity; TV: television

2. Variables that had a high unexplained variance (> 0.60) were removed from the principal components analysis.

3. These variables were excluded due to the lack of variation in responses rather than having a high unexplained variance.

One variable in the parental support construct had a high unexplained variance suggesting that it did not fit with the other parent support variables and should be analysed as an individual variable. The remaining items in the parental support construct, however, all loaded onto one factor which had good internal consistency. Two maternal self-efficacy variables and two barriers variables were also excluded from the PCA on these constructs due to having a high unexplained variance. The remaining variables in each of these constructs were found to load onto one factor and both had acceptable internal consistency.

For the questions on parental restrictions, those relating to restricting children playing outside and restricting children's television viewing were found to have a high unexplained variance and therefore were not part of the rest of the construct. Removal of these variables made a PCA analysis on the remaining 'restriction' variables redundant as only two variables remained. These variables combined had acceptable internal consistency (see Table 4).

The questions on parental rules were initially found to load onto three factors, however, the third factor had a low Cronbach's alpha (α = 0.16). One of the variables in the third factor was found to have little variation in responses (88.8% were in one answer category) and this is likely to have contributed to the low Cronbach's alpha. Re-running the analysis without this variable produced the same three factors, with the third factor now only containing a single variable. These results suggest that a two-factor structure is more appropriate for these questions in this dataset with the variable in the third factor being treated as an individual item. Similar results were found for the questions on the local environment. Although three factors were initially identified, the third factor had a low Cronbach's alpha (α = 0.12) and was found to contain a variable with limited variability (87.2% were in one answer category). When analyses were re-run without this variable, a two-factor structure emerged, with the remaining variable from the original third factor having a high unexplained variance by these two factors (see Table 4). This suggests that the latter should be treated as a separate variable in analyses.

## Discussion

Valid and reliable measures of physical activity correlates in young children are lacking but are needed if we are to understand which factors might be usefully targeted in interventions to promote physical activity in this age group. The studies presented in this paper investigated the validity, factor structure and internal consistency of a maternal questionnaire on the correlates of physical activity in four-year-old children. The first study showed that there was reasonable agreement between most of the questionnaire items and responses to the same or similar questions given during telephone interviews, providing some evidence that the questionnaire is valid. Further evidence of validity was shown from the PCA in Study II which identified eight factors with acceptable internal consistency that reflected different constructs within the questionnaire. Together these results suggest that, with some minor revisions, this questionnaire could be a useful tool for measuring potential correlates of preschool children's physical activity.

### Child personal factors

Despite the mixed kappa scores, the agreement between the questionnaire and interview responses regarding child personal factors was generally good indicating reasonable validity of these questions. The one question in this domain that performed less well was the question regarding whether children preferred to watch television or play with their toys. Several interview participants commented that their child often did these activities at the same time, making it difficult to answer this question. The question might therefore need to be rephrased in some way to reflect this. This question has previously been used in older children [30] and it might be more appropriate in older age groups where children are able to report their own preferences. The PCA identified two factors from the questions on child personality and enjoyment of physical activity while whether or not mothers described their child as outgoing did not seem to be part of either factor. It may be that this item should stand alone in analyses but it is also possible that the inclusion of more items within the personality construct might show that this individual variable is part of another composite factor not addressed by the current questions.

### Parental influences

The main focus of the questionnaire was on potential parental influences on preschool children's physical activity. The majority of these questions had high agreement between the interview and questionnaire responses with the low kappa scores tending to correspond to variables with little variation. These results provide good evidence of the validity of these items. Four items, however, had poor validity. The low agreement for the question on mothers' self-efficacy for taking their child out to play when it was cold and wet may be explained by the participants who commented in the interview that this question was not really applicable as they would not take their child out to play in this weather. The question therefore perhaps needs to be rephrased to only cover cold weather rather than cold and wet weather. The poor agreement for mothers' self-efficacy for playing an active game with their child on a busy day was due to participants reporting greater difficulty in doing this in the questionnaire than during the interview, however, it is not clear why this was the case. The question is fairly broad therefore perhaps revising it to be more specific could help improve its validity. The revision of the question relating to whether children were allowed to run or cycle in the house to whether children were allowed to run or ride a tricycle in the house, based on feedback from the interviews, is likely to have improved the validity of this question. Similarly, the addition of a non-applicable option to the questions on restricting children's computer use is thought to have increased the validity of these questions.

The finding that the questions on parental support generally reflected one construct is supported by findings from Saunders et al [20] who identified one factor representing both parent and peer support in a principal components analysis of a questionnaire designed for fifth grade (10-11 year-old) children. A confirmatory factor analysis by Ommundsen et al [41], however, suggested that parental support questions were better divided into two factors: parental encouragement and parental social support. The sample in this latter study consisted of both children (aged 9-10 years) and adolescents (aged 15-16 years) and it may be that the factor structure of these questions differs for younger children. Previous research on the association between parental support and preschool children's physical activity has mostly focused specifically on parental encouragement and persuasion [10]. Further research is therefore needed on the factor structure of a broader range of parental support questions relating to preschool children to confirm the findings reported in the analysis in this paper.

A single factor also emerged from the PCA of the questions on maternal self-efficacy for social support, however, two questions had a high unexplained variance by this factor suggesting that they do not belong to the same construct. Some studies that have investigated the factor structure of questions on children's or adolescents' self-efficacy for physical activity have reported that these questions load onto one factor [19,42] while one study that included a greater number of questions (17 compared with < 10) reported a three-factor structure for self-efficacy questions [20]. It may therefore be the case that the two maternal self-efficacy questions in this analysis found to have a high unexplained variance by the factor structure identified might reflect other factors relating to maternal self-efficacy that more questions would help uncover.

The parental rules questions were found to load onto three underlying factors, two relating to indoor rules and one relating to outdoor rules (consisting of one variable). Sallis et al [43] reported independent associations between indoor and outdoor rules and preschool children's physical activity, supporting the breakdown of parental rules into more than one factor. However, the fact that two of the rules factors in this analysis related to indoor rules suggests that the rules construct can be broken down further. This implies that different types of rules may reflect different constructs and these could potentially be differentially associated with preschool children's physical activity. It is therefore important that rules-related questions are not simply combined into one composite factor as this could result in associations with physical activity being missed. Similar results were found for the questions on parental restrictions, with restrictions on television watching, computer use and computer game playing, and playing outside being identified as three separate variables, respectively.

### Barriers to preschool children's physical activity

The barriers questions all had relatively low kappa scores, ranging from poor to fair, but the percentage agreement was generally high providing evidence that these questions are reasonably valid. The finding that these barriers questions reflected several underlying factors is supported by the results of a confirmatory factor analysis of a questionnaire on psychosocial correlates of physical activity in adolescents which also found barriers to physical activity to consistent of several different factors [17]. As with parental rules and restrictions, this suggests that there are different types of barriers to physical activity which may be differentially associated with children's physical activity and should therefore be assessed as separate constructs.

### Home and local environment

All of the home environment questions had good agreement between the interview and questionnaire responses indicating good validity. The local environment questions also generally had good agreement but one question regarding concern about 'stranger danger' was found to have poor validity. Some participants said in the interview that 'stranger danger' was not a concern to them as their child never went out without an adult. However, most of these participants indicated in the questionnaire that stranger danger was a concern, possibly suggesting that their responses to the questionnaire were more about general concerns while in the interview they responded specifically about their concern for their child. There may also have been some effect of social norms where participants felt that they should be concerned about strangers but felt comfortable expressing no concern about strangers in the interview when they could explain their reasons for this. Rewording this question or the response categories to this question in light of this could therefore help improve its validity.

The finding that different aspects of the local environment reflect different constructs is supported by the results of a study of 11-year-old children's perception of the physical activity environment, which found good internal consistency for scores on neighbourhood safety and the neighbourhood social environment [18]. This emphasises the need to have a range of questions that cover different potential influences in the local environment so that all underlying constructs are captured.

### Limited variation

An issue that arose in both studies presented in this paper was the limited variation in responses for some variables. In the PCA, this issue affected the Cronbach's alpha of some of the factors identified and may have impacted on the factor loadings for the variables with limited variation. In the validity assessment, this issue is likely to have contributed to some of the low kappas[37], such as those for whether there is a garden at home where the child can play (88% of interview participants answered positively), car ownership (100% had a car), and whether the child's physical activity is important (91% of interview participants answered positively). As a result, greater weight was given to the percentage agreement between the interview and questionnaire responses than to the kappa values. Although kappa statistics are generally preferred over percentage agreement as they reflect the level of agreement after taking chance into account, the low variability in responses in this dataset limits the stability of the kappa statistic. Basing the results of the validity analysis on the kappa values may therefore be misleading.

### Strengths and Limitations

The validation of measures of physical activity correlates in children is hampered by the lack of a 'gold standard' with which to compare the new measure. One option is to compare the questionnaire responses to a physical activity outcome therefore measuring the predictive validity of the questionnaire. However, we felt that this approach was not appropriate for the current study as our aim was to assess how well the questionnaire measured the *potential *correlates of physical activity that we were trying to measure, not assess whether these questionnaire items were indeed associated with children's physical activity. We therefore chose to investigate the criterion validity of the questionnaire by comparing the questionnaire responses to responses given in a telephone interview. Interviews have previously been used to validate questionnaires that assess health states or behaviours [44,45] and the literature on qualitative interviewing highlights the use of interviews to gain more in-depth responses than those achieved from survey questions. Using telephone interviews as the criterion measure was therefore a strength of this study as they gave greater insight into participants' answers and provided important feedback, aiding the revision of questions that performed less well in the analyses. However, there are some limitations of this validation approach, such as the questionnaire and interview data coming from the same source making it likely that there is correlated error between the two measures. Also, there is the possibility of interviewer and interpretation bias, although attempts were made to minimise the former by conducting two pilot interviews and the latter by double-coding the interview responses, independently of the questionnaire responses.

The large number and variety of potential correlates covered in the questionnaire being validated were strengths of this study. It is important to acknowledge that the sample for the validity assessment was small and not fully representative of the larger sample in Study II. The larger sample used in Study II, however, was comparable to the rest of the four-year SWS sub-sample seen for a visit before March 2006 and to the population of Southampton as a whole in terms of ethnicity. This implies that the results of the studies are applicable to the target study population.

## Conclusion

This paper has shown that a maternal questionnaire on the correlates of young children's physical activity has reasonable validity overall and acceptable internal consistency for most of the factors identified from the principal components analysis. With some minor revisions, this questionnaire might be a useful tool for investigating physical activity correlates in young children in populations with similar characteristics to the study population. Further analyses are needed to show how generalisable these results are to other populations and to explore whether the factors measured by the questionnaire are associated with preschool children's physical activity.

## Competing interests

The authors declare that they have no competing interests.

## Authors' contributions

AMM was involved in the study design and the recruitment of participants to Study I, conducted the telephone interviews in Study I, performed the statistical analyses for both studies, and was responsible for drafting and revising the manuscript. EMFvS designed the questionnaire and both EMFvS and SJG provided supervision on the study design, data collection and statistical analyses, and were involved in revising the manuscript. NCH, CC, HMI, and KMG all made substantial contributions to the design and conduct of the Southampton Women's Survey and were involved in revising the manuscript. Additionally, NCH was responsible for the distribution of the questionnaire to SWS participants. All authors read and approved the final manuscript.

## Supplementary Material

Additional file 1 **SWS questionnaire**. This file contains a copy of the revised version of the questionnaire used in Study II. Click here for file
