# Supplementary material for: Validation of a maternal questionnaire on correlates of physical activity in preschool children
Source: Int J Behav Nutr Phys Act. 2009 Dec 2;6:81. doi: 10.1186/1479-5868-6-81 (PMC2791748; doi:10.1186/1479-5868-6-81)
Supplement: Additional file 1 — SWS questionnaire. This file contains a copy of the revised version of the questionnaire used in Study II. [file 1479-5868-6-81-S1.DOC]

**SWS Serial Number**


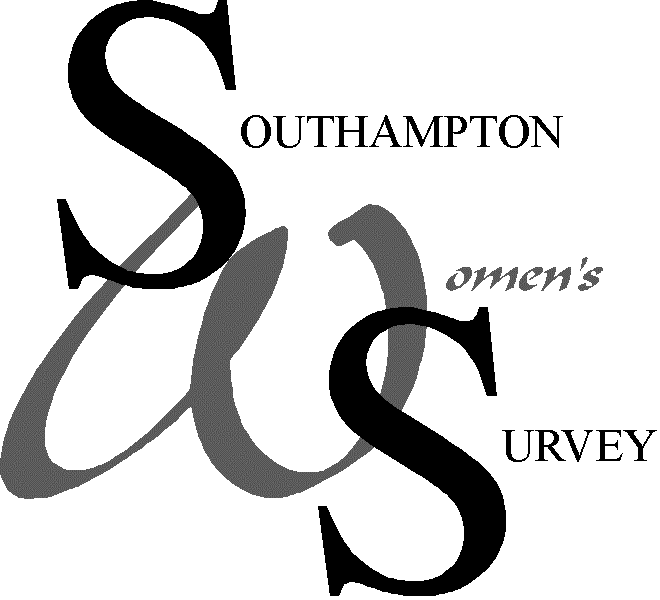


Questionnaire on physical activity

4-year old child - v 1.3

* Completing this questionnaire will take 15 to 20 minutes

* Please answer all the questions as best as you can

* If you have any questions, please phone our freephone number 0800 783 4503 and ask to speak to a member of the research team about the physical activity questionnaire

#### * Your answers will be treated as confidential

This questionnaire asks for some background information about you, your physical activity and your child’s activity. **Please note:** All the questions in this questionnaire relate to **your 4-year old child** who is being measured as part of the Southampton Women’s Survey (SWS).

| Section 1. Information about yourself and your household |
| --- |

**1.** Please write down today’s date (dd/mm/yy): ………… / ………… / ………………

**2.** What is **your** date of birth (dd/mm/yy)? ………… / ………… / ………………

**3.** At what age did you finish continuous full time education? ………………… years

**4.** Do you own or rent your home?

|  | Own it/buying it |
| --- | --- |
|  |  |
|  | Rent it |

**5.** To which of these groups do **you** consider **you** belong? *(please tick one box)*

|  | White |
| --- | --- |
|  |  |
|  | Black, Caribbean |
|  |  |
|  | Black, African |
|  |  |
|  | Black, other (please give details): ………………………………………………………… |
|  |  |
|  | Indian |
|  |  |
|  | Pakistani |
|  |  |
|  | Bangladeshi |
|  |  |
|  | Chinese |
|  |  |
|  | Other (please give details): …………………………………………………………………… |

**6.** What is your home postcode?

|  |  |  |  |  |  |  |
| --- | --- | --- | --- | --- | --- | --- |

**7.** What is **your** height?

|  | ft |  |  | inch |
| --- | --- | --- | --- | --- |
|  |  |  |  |  |
|  | m |  |  | cm |

OR

**8.** What is **your** current weight?

|  |  | st |  |  | Lb |
| --- | --- | --- | --- | --- | --- |
|  |  |  |  |  |  |
|  |  |  | kg |  |  |

OR

# The following questions are about your household

|  | Yes |
| --- | --- |
|  |  |
|  | No |

**9.** Do you live together with the father of your 4-year old?

|  | Yes |
| --- | --- |
|  |  |
|  | No |

**10.** Are there any other adults living in your home?

If yes: please state their relationship to your child (e.g. stepfather, grandmother):

…………………………………………………………………………………………………………………………………………

**11.** How many children **younger** than your 4-year old live in your household? …………….

**12.** How many children **older** than your 4-year old live in your household? ………………….

**Answer categories for question 13:**

1. strongly disagree
2. disagree
3. neither disagree nor agree
4. agree
5. strongly agree

| 1. Do you agree or disagree with the following statements about your home environment? | Strongly  disagree | | Strongly  agree | |
| --- | --- | --- | --- | --- |
| 1. There is heavy traffic in our local streets. 2. I am concerned about ‘stranger danger’. 3. There is somewhere at home where my child can go out and play (such as a garden). 4. I am concerned about road safety in our area. 5. Public transport is limited in our area. 6. There are play areas, parks, or gyms close to our home where my child can play. 7. There are other children near our home my child can play with. 8. At my child’s nursery/day care there are playgrounds or fields where my child can run around (if applicable). | | 1 2 3 4 5  1 2 3 4 5  1 2 3 4 5  1 2 3 4 5  1 2 3 4 5  1 2 3 4 5  1 2 3 4 5  1 2 3 4 5 | |  |
| □ *not applicable, child not at nursery or day care* | | | |  |

**14.** Does your household have any cars or vans normally

|  | Yes |
| --- | --- |
|  |  |
|  | No |

available for its use?

**15.** Does your child attend day care, nursery or preschool? *(please tick one box)*

|  | Yes, full time (30 hours or more per week) |
| --- | --- |
|  |  |
|  | Yes, part time (less than 30 hours per week) |
|  |  |
|  | No, he/she is at home with me |
|  |  |
|  | No, he/she is at home with another adult |
|  |  |
|  | Other, (please state): ………………………………………………………………… |

**16.** Does your child have any of the following in his/her own bedroom?

| a. TV |  | Yes |  | No |
| --- | --- | --- | --- | --- |
|  |  |  |  |  |
| b. Video or DVD player |  | Yes |  | No |
|  |  |  |  |  |
| c. Computer (PC, Playstation/Xbox) |  | Yes |  | No |

| Section 2. Your own activities |
| --- |

1. On average over the last 4 weeks, how many hours did **you** spend on TV or video viewing, and on computer use at home:

## a. TV viewing or video watching

(Please put one tick (✓) on every line)

| Hours of **TV or video watched** per day | **Average over the last 4 weeks** | | | | | |
| --- | --- | --- | --- | --- | --- | --- |
|  | None | Less than 1 hour a day | 1 to 2 hours a day | 2 to 3 hours a day | 3 to 4 hours a day | More than 4 hours a day |
| On a weekday |  |  |  |  |  |  |
| On a weekend day |  |  |  |  |  |  |

## b. Computer use

*At home but not at work, such as internet, email, Playstation, Xbox, Gameboy.*

(Please put one tick () on every line)

| Hours of **home computer use** per day | **Average over the last 4 weeks** | | | | | |
| --- | --- | --- | --- | --- | --- | --- |
|  | None | Less than 1 hour a day | 1 to 2 hours a day | 2 to 3 hours a day | 3 to 4 hours a day | More than 4 hours a day |
| On a weekday |  |  |  |  |  |  |
| On a weekend day |  |  |  |  |  |  |

1. When making short trips **alone**, what form of transport do you **usually** use? (by a short trip we mean less than ½ mile) *(please tick one box)*

|  | Public transport |
| --- | --- |
|  |  |
|  | Car |
|  |  |
|  | Walking |
|  |  |
|  | Bicycling |
|  |  |
|  | Other, (please state): ………………………………………………………………… |

1. When making short trips **with your child(ren)**, what form of transport do you **usually** use? (by a short trip we mean less than ½ mile) *(please tick one box)*

|  | Public transport |
| --- | --- |
|  |  |
|  | Car |
|  |  |
|  | Walking |
|  |  |
|  | Bicycling |
|  |  |
|  | Other, (please state): ………………………………………………………………… |

1. We would like to know the type and amount of physical activity involved in your work. Please **tick one option** that best corresponds to your present activities from the following five possibilities.

|  | Not in employment |
| --- | --- |
|  | You are for example retired, unemployed, or a full-time carer |
|  | Sedentary occupation |
|  | You spend most of your time sitting (such as in an office) |
|  | Standing occupation |
|  | You spend most of your time standing or walking. However, your work does not require intense physical efforts (e.g. shop assistant, hairdresser, guard etc) |
|  | Physical work |
|  | This involves some physical effort including handling of heavy objects and use of tools (e.g. plumber, cleaner, nurse, sports instructor, electrician, carpenter, etc) |
|  | Heavy manual work |
|  | This involves very vigorous physical activity including handling of very heavy objects (e.g. docker, miner, bricklayer, construction worker etc) |

1. In a typical week during the past 12 months, how many hours did **you** spend on each of the following activities? (Put '0' if none)

- Walking, including walking to work, shopping and leisure

In summer ____ hours per week

In winter ____ hours per week

- Cycling, including cycling to work and during leisure time

In summer ____ hours per week

In winter ____ hours per week

- Other physical exercise such as keep fit, aerobics, swimming, jogging

In summer ____ hours per week

In winter ____ hours per week

| Section 3. Your child’s activities |
| --- |

1. When you compare your 4-year old child to your other child(ren), would you say that *(please tick one box)*:

|  | Not applicable, I do not have other children |
| --- | --- |
|  |  |
|  | He/she is as active as my other child(ren) |
|  |  |
|  | He/she is more active then my other child(ren) |
|  |  |
|  | He/she is less active then my other child(ren) |

1. Compared with children from the same age group and the same sex, I would say that my child is *(please tick one box)*:

|  | Generally less active |
| --- | --- |
|  |  |
|  | Similarly active |
|  |  |
|  | Generally more active |

1. In general, would your child’s own preference be to *(please tick one box per line)*:

| play indoors |  | OR |  | play outdoors |
| --- | --- | --- | --- | --- |
|  |  |  |  |  |
| play with toys |  | OR |  | watch TV |
|  |  |  |  |  |
| watch TV |  | OR |  | playing a running game with siblings or friends |

| Section 4. Your view on your child’s activity |
| --- |

The questions in this section ask about your views on aspects of your 4-year old’s physical activity behaviour.

**Answering questions 1 to 3**

Please circle one answer per question, using the answer categories below.

1. strongly disagree
2. disagree
3. neither disagree nor agree
4. agree
5. strongly agree

| 1. Would you describe your child as: | Strongly  disagree | | Strongly  agree | |
| --- | --- | --- | --- | --- |
| - 1. physically active   2. restless   3. well-behaved   4. outgoing | | 1 2 3 4 5  1 2 3 4 5  1 2 3 4 5  1 2 3 4 5 | |  |

| 1. Do you agree or disagree with the following statements about your child’s activity? | Strongly  disagree | | Strongly  agree | |
| --- | --- | --- | --- | --- |
| 1. My child enjoys being physically active 2. I am concerned about the amount of TV my child watches    1. I think it is important that my child participates in physical activity and/or sports | | 1 2 3 4 5  1 2 3 4 5  1 2 3 4 5 | |  |

| 1. I think it is difficult… | Strongly  disagree | | Strongly  agree | |
| --- | --- | --- | --- | --- |
| - 1. …to encourage my child to go outside and play   2. …to encourage my child to play an active game instead of watching TV   3. …to play an active game with my child on a busy day   4. …to take my child outside to play when it is cold and wet outside   5. …to take my child outside to play when it is hot outside   6. …to play an active game with my child at the weekend   7. …to play an active game with my child when I am tired | | 1 2 3 4 5  1 2 3 4 5  1 2 3 4 5  1 2 3 4 5  1 2 3 4 5  1 2 3 4 5  1 2 3 4 5 | |  |

**Answering questions 4 to 7**

Please circle one answer per question, using the answer categories below.

1. never
2. rarely
3. sometimes
4. often
5. very often

| 1. In general, how often do you or your partner… | Never | | Very  often | |
| --- | --- | --- | --- | --- |
| - 1. Encourage your child to do physical activities or play sports?   2. Do a physical activity or play sports with your child?   3. Provide transport so your child could go to a place where he/she can do physical activities or play sports?   4. Watch your child participate in physical activity or sport?   5. Tell your child that being physically active is good for his/her health? | | 1 2 3 4 5  1 2 3 4 5  1 2 3 4 5  1 2 3 4 5  1 2 3 4 5 | |  |

| 1. In general, how often do you or your partner allow your child to do the following? | Never | | Very  often | |
| --- | --- | --- | --- | --- |
| 1. Watch TV at meal times 2. Go to bed when they want to 3. Play ball games in the house 4. Eat snacks while watching TV 5. Play in the park/ play area accompanied by older children (without adult supervision) 6. Run or ride a tricycle/scooter in the house    1. Play in the garden without adult supervision | | 1 2 3 4 5  1 2 3 4 5  1 2 3 4 5  1 2 3 4 5  1 2 3 4 5  1 2 3 4 5  1 2 3 4 5 | |  |

| 1. In general, how often do you or your partner restrict the time your child spends doing the following activities? | Never | | Very  often | *Not appli-cable* | |
| --- | --- | --- | --- | --- | --- |
| 1. Watching TV/video 2. Playing computer games (such as Xbox, PlayStation) 3. Playing outside 4. Using the computer | | 1 2 3 4 5 *6*  1 2 3 4 5 *6*  1 2 3 4 5 *6*  1 2 3 4 5 *6* | | |  |

| 1. How often is **your child limited from doing an activity** because: | Never | Very  often | |
| --- | --- | --- | --- |
| 1. The fees for clubs or swimming pools are too high 2. It is difficult to get to physical activity places 3. My child doesn’t have the skills to do the activity 4. My child is not interested in the activity 5. The weather is too bad 6. I am too busy 7. I am scared that my child will get hurt 8. There are no play areas/parks near our home 9. There are no other children to play with 10. There is no adult to supervise the child whilst playing | 1 2 3 4 5  1 2 3 4 5  1 2 3 4 5  1 2 3 4 5  1 2 3 4 5  1 2 3 4 5  1 2 3 4 5  1 2 3 4 5  1 2 3 4 5  1 2 3 4 5 | |  |

**Thank you for completing this questionnaire**

**Remarks about this questionnaire**

*Please give us your comments, such as any questions you thought were difficult to understand or where it was not clear how to answer the question.*

##### Please return the questionnaire to the SWS office in the pre-paid envelope along with the ActiHeart activity monitor
